# Prediction of Two Molecular Subtypes of Gastric Cancer Based on Immune Signature

**DOI:** 10.3389/fgene.2021.793494

**Published:** 2022-01-17

**Authors:** Dan Wu, Mengyao Feng, Hongru Shen, Xilin Shen, Jiani Hu, Jilei Liu, Yichen Yang, Yang Li, Meng Yang, Wei Wang, Qiang Zhang, Fangfang Song, Ben Liu, Kexin Chen, Xiangchun Li

**Affiliations:** ^1^ National Clinical Research Center for Cancer, Key Laboratory of Cancer Prevention and Therapy, Tianjin Cancer Institute, Tianjin Medical University Cancer Institute and Hospital, Tianjin Medical University, Tianjin, China; ^2^ Department of Epidemiology and Biostatistics, Key Laboratory of Molecular Cancer Epidemiology of Tianjin, National Clinical Research Center for Cancer, Key Laboratory of Cancer Prevention and Therapy, Tianjin Medical University Cancer Institute and Hospital, Tianjin Medical University, Tianjin, China; ^3^ Department of Maxillofacial and Otorhinolaryngology Oncology, Tianjin Medical University Cancer Institute and Hospital, Tianjin Medical University, Tianjin, China

**Keywords:** gastric cancer, immune signature, molecular subtypes, prognosis, computational biology

## Abstract

Gastric cancer is the fifth most common type of human cancer and the third leading cause of cancer-related death. The purpose of this study is to investigate the immune infiltration signatures of gastric cancer and their relation to prognosis. We identified two distinct subtypes of gastric cancer (C1/C2) characterized by different immune infiltration signatures. C1 is featured by immune resting, epithelial–mesenchymal transition, and angiogenesis pathways, while C2 is featured by enrichment of the MYC target, oxidative phosphorylation, and E2F target pathways. The C2 subtype has a better prognosis than the C1 subtype (HR = 0.61, 95% CI: 0.44–0.85; log-rank test, *p* = 0.0029). The association of C1/C2 with prognosis remained statistically significant (HR = 0.62, 95% CI: 0.44–0.87; *p* = 0.006) after controlling for age, gender, and stage. The prognosis prediction of C1/C2 was verified in four independent cohorts (including an internal cohort). In summary, our study is helpful for better understanding of the association between immune infiltration and the prognosis of gastric cancer.

## Introduction

Gastric cancer (GC) ranks the fifth most commonly diagnosed cancer type globally and the third leading cause for cancer-related death, which was attributed to its diagnosis usually made at an advanced stage. Although gastric cancer incidence has declined in most countries over the past century, the aging population may contribute to increased diagnosis of gastric cancer (Smyth et al., 2020).

Gastric cancer is a highly heterogeneous disease characterized by histopathologic and epidemiologic features based on molecular and phenotypic levels (Van Cutsem et al., 2016). Next-generation sequencing has showed new insights into the heterogeneity of gastric cancer, and subtyping systems have been proposed (Cancer Genome Atlas Research, 2014; Cristescu et al., 2015; Sohn et al., 2017; Kim et al., 2019; Zhang et al., 2020). The Lauren classification system categorizes gastric cancer into the intestinal and diffuse subtypes (Lauren, 1965), while the WHO system divides gastric cancer into four subtypes (papillary, tubular, mucinous, and poorly cohesive) (Hu et al., 2012). Apart from the aforementioned classification subtypes, researchers from TCGA proposed four subtypes for gastric cancer: Epstein–Barr virus (EBV) positive, microsatellite unstable (MSI), genomically stable (GS), and chromosomal instability (CIN). The EBV subtype has the best prognosis among these four subtypes. Patients with the CIN subtype experienced the greatest benefit from adjuvant chemotherapy (Sohn et al., 2017). The Asia Cancer Research Group (ACRG) proposed four molecular subtypes for GC based on microsatellite instability, epithelial–mesenchymal transition, and TP53 mutation: MSI, MSS/EMT, MSS/TP53^+^, and MSS/TP53^-^ (Cristescu et al., 2015). The EBV subtype and MSI subtype were reported to potentially benefit from immunotherapy (Pang et al., 2009; Le et al., 2017; Amatatsu et al., 2018; Sundar et al., 2018). However, few gastric cancer subtypes development is based on immune signature and can be used to predict the prognosis of gastric cancer patients.

Immune processes play critical roles in carcinogenesis and progression of solid tumors, and they also affect the treatment and prognosis of patients. Researchers are confused with the association between the immune environment and the prognosis, and much attention has been paid to the tumor immune environment (Cully, 2018; Lim et al., 2018; Kubota et al., 2021). All tumors are potentially immunogenic, and the new knowledge about the interactions between tumor cells, immune cells, and tumor microenvironment allowed for reversal of possible immune resistance (Refolo et al., 2020; Ceresoli and Pasello, 2021). The immune response is a complex multistep process that finely regulates the balance between the recognition of non-self and the prevention of autoimmunity. Cancer cells can use these pathways to suppress tumor immunity as a major mechanism of immune resistance. The recent molecular classifications of gastric cancer by The Cancer Genome Atlas (TCGA) and by the Asian Cancer Research Group (ACRG) networks, together with the identification of multiple biomarkers, open new perspectives for stratification of patients who might benefit from a long-term immune checkpoint therapy (Newman et al., 2015; Jiang et al., 2018; Thorsson et al., 2019; Ceresoli and Pasello, 2021).

The purposes of this study are to characterize different potential molecular classification systems operative in gastric cancer and to identify previously unreported significant immune environments and independent prognosis factors for patients with gastric cancer. We collected 1386 samples from five datasets and applied molecular subtyping on each dataset. We achieved two distinct molecular subtypes of gastric cancer (i.e., C1/C2). The C2 subtype has a better prognosis and more activated immune microenvironment than the C1 subtype.

## Methods

### Data Collection and Processing

In total, our study included five gastric cancer datasets: TCGA dataset, three datasets from Gene Expression Omnibus (i.e., GSE62254, GSE15459, and GSE84437), and one internal dataset. TCGA dataset was used as discovery set, whereas the other four datasets as validation sets. The expression matrix of each dataset was normalized individually. The 3151 immune-related genes were collected from previous studies (Supplementary Table S1) (Thorsson et al., 2019). We applied removeBatchEffect from the R *limma* package (version 3.34.9) to remove the batch effect while combining these five datasets.

### Consensus Molecular Subtyping

We conducted survival analysis in TCGA dataset. Genes significant in survival analysis from TCGA dataset were reversed in follow-up molecular subtyping. We applied consensus non-negative matrix factorization (CNMF) clustering for finding molecular patterns from high-dimensional biological datasets. It is combined with a quantitative evaluation of the robustness of the number of clusters. The CNMF method is included in *CancerSubtypes* (version 1.18.0), an R package for clustering cancer subtypes (Brunet et al., 2004; Xu et al., 2017).

### Survival Analysis and Multivariable Cox Regression Analysis

The association of C1/C2 with overall survival was estimated using Kaplan–Meier plots and log-rank tests. Multivariable Cox regression analysis was used to evaluate independent prognostic factors associated with overall survival, including age, gender, and tumor stage. A *p* value of less than 0.05 was considered statistically significant.

### Statistical Analysis

Differentially expressed genes (DEGs) between C1 and C2 were evaluated by *edgeR* package (version 3.28.1). The *clusterProfiler* package (version 3.14.3) was used for pathway enrichment.

The CIBERSORT method was used to characterize the composition of 22 kinds of immune cells from RNA expression (Newman et al., 2015). The t-test was used for comparing the CIBERSORT score, and a *p* value of less than 0.05 was considered statistically significant.

The mutation of significantly mutated genes (SMGs) in C1/C2 was compared in this part (Li et al., 2016). The chi-square test was used for comparing the proportion of some somatic mutations in between C1 and C2. A *p* value of less than 0.05 was considered statistically significant.

Tumor mutational burden (TMB) was calculated as the number of mutation events per sample. The t-test was used for comparing the CIBERSORT score, and a *p* value of less than 0.05 was considered statistically significant. All statistical analyses were done by R software (version 3.4.3).

## Results

### Patients and Clinical Information

**FIGURE 1:**
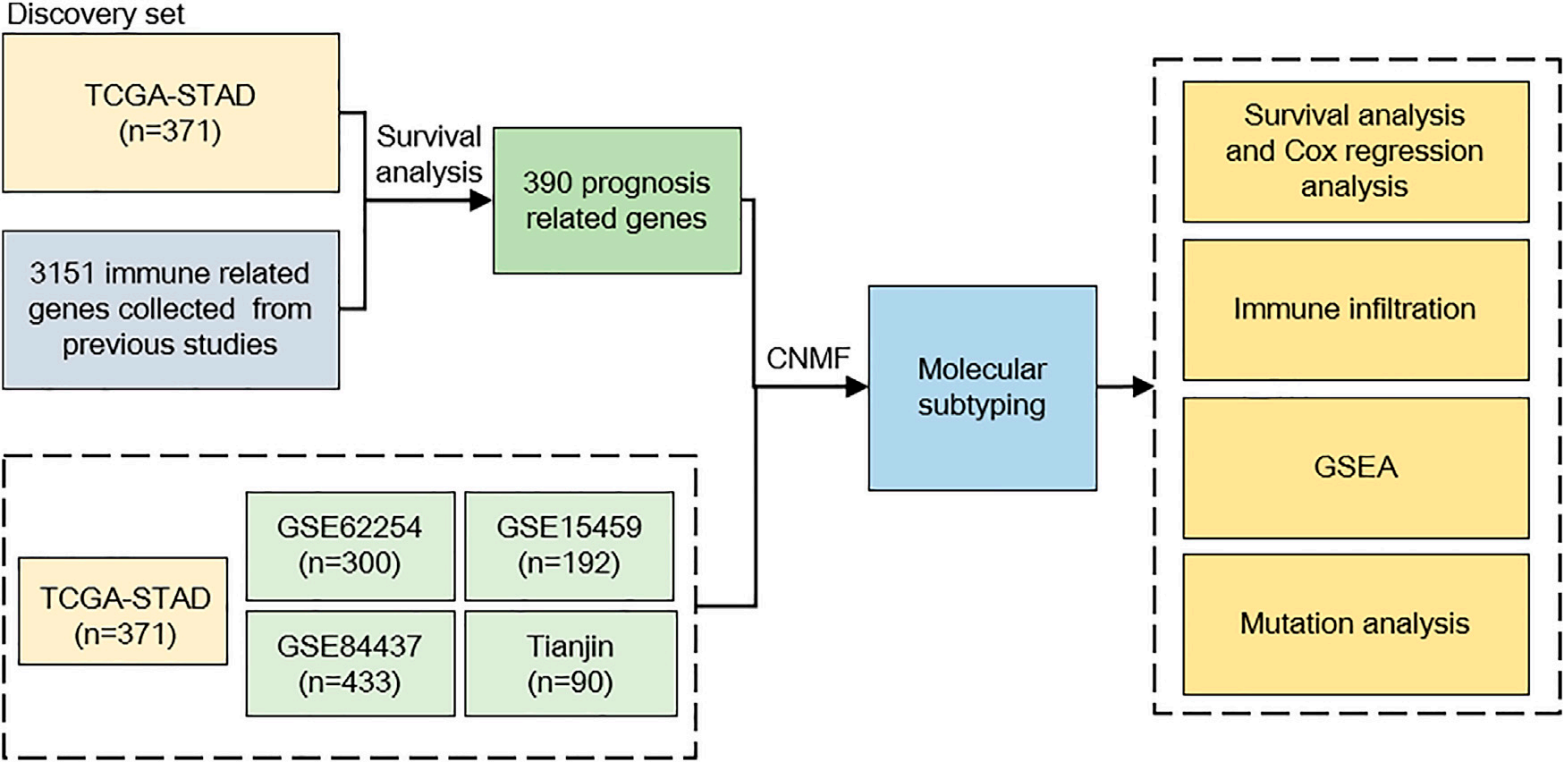
Study flowchart. We incorporated 1386 gastric cancer (GC) samples in this study. TCGA cohort was used as a discovery set; after evaluating prognosis-related genes, 390 genes were selected to predict GC subtypes. The CNMF model was used to develop GC subtypes on five cohorts; after molecular subtyping, we did survival analysis, immune infiltration analysis, GSEA analysis, and mutation analysis to describe different characteristics between our subtypes. CNMF, consensus non-negative matrix factorization; GSEA, gene set enrichment analysis.

The study flowchart was shown in Figure 1. In total, we collected 1386 gastric cancer samples from five datasets. These five datasets include 371 samples from TCGA cohort, 300 from GSE62254, 192 from GSE15459, 433 from GSE84437, and an internal dataset of 90 samples (Tianjin cohort). Patients’ stages ranged from stage 1 to stage 4 (stage 1: 9.5%; stage 2: 28.8%; stage 3: 44.2%; stage 4:15.8%), and all these five cohorts have clinical information including overall survival (OS), vital stage, age, gender, and tumor stage. In addition to this, GSE62254 cohort and GSE15459 cohort also has Lauren type information. In the GSE62254 cohort, 150 (50%) patients are intestinal type, 142 (47%) patients are diffuse type, and 8 (3%) patients are mixed type. In the GSE15459 cohort, 75 (38%) patients are intestinal type, 99 (52%) patients are diffuse type, and 18 (9%) patients are mixed type. The basic information of these cohorts is reported in Table 1.

**TABLE 1:** Baseline characteristics.

	TCGA^a^ (*n* = 371)	GSE62254 (*n* = 300)	GSE15459 (*n* = 192)	GSE84437 (*n* = 433)	Tianjin (*n* = 90)
Gender					
Male	238 (64%)	199 (67%)	125 (65%)	296 (68%)	59 (32%)
Female	133 (36%)	101 (33%)	67 (35%)	137 (32%)	34 (68%)
Age	68 (36–90)	64 (24–86)	67 (23–92)	62 (27–86)	58 (33–87)
Age ≤60 years male	78 (21%)	72 (24%)	31 (16%)	130 (30%)	28 (31%)
Age >60 years male	156 (42%)	127 (42%)	94 (49%)	166 (38%)	31 (35%)
Age ≤60 years female	31 (8%)	45 (15%)	28 (15%)	64 (15%)	21 (23%)
Age >60 years female	100 (27%)	56 (19%)	39 (20%)	73 (17%)	10 (11%)
Stage					
1	50 (14%)	30 (10%)	31 (16%)	21 (5%)	0
2	111 (30%)	97 (32%)	29 (15%)	138 (32%)	24 (27%)
3	149 (40%)	96 (32%)	72 (38%)	274 (63%)	22 (24%)
4	38 (10%)	77 (26%)	60 (31%)	0	44 (49%)
Missing	23 (6%)	0	0	0	0
Lauren type					
Intestinal	NA	150 (50%)	75 (38%)	NA	NA
Diffuse	NA	142 (47%)	99 (52)	NA	NA
Mixed	NA	8 (3%)	18 (10%)	NA	NA
Survival time (years)	1.21 (0–5)	4.82 (0.08–8.81)	1.58 (0–13.15)	5.75 (0–13.42)	2.62 (0–13.81)

a In TCGA cohort, six samples (2%) have no age information.

### Classification of Gastric Cancer Molecular Based on Immune Genes

**FIGURE 2:**
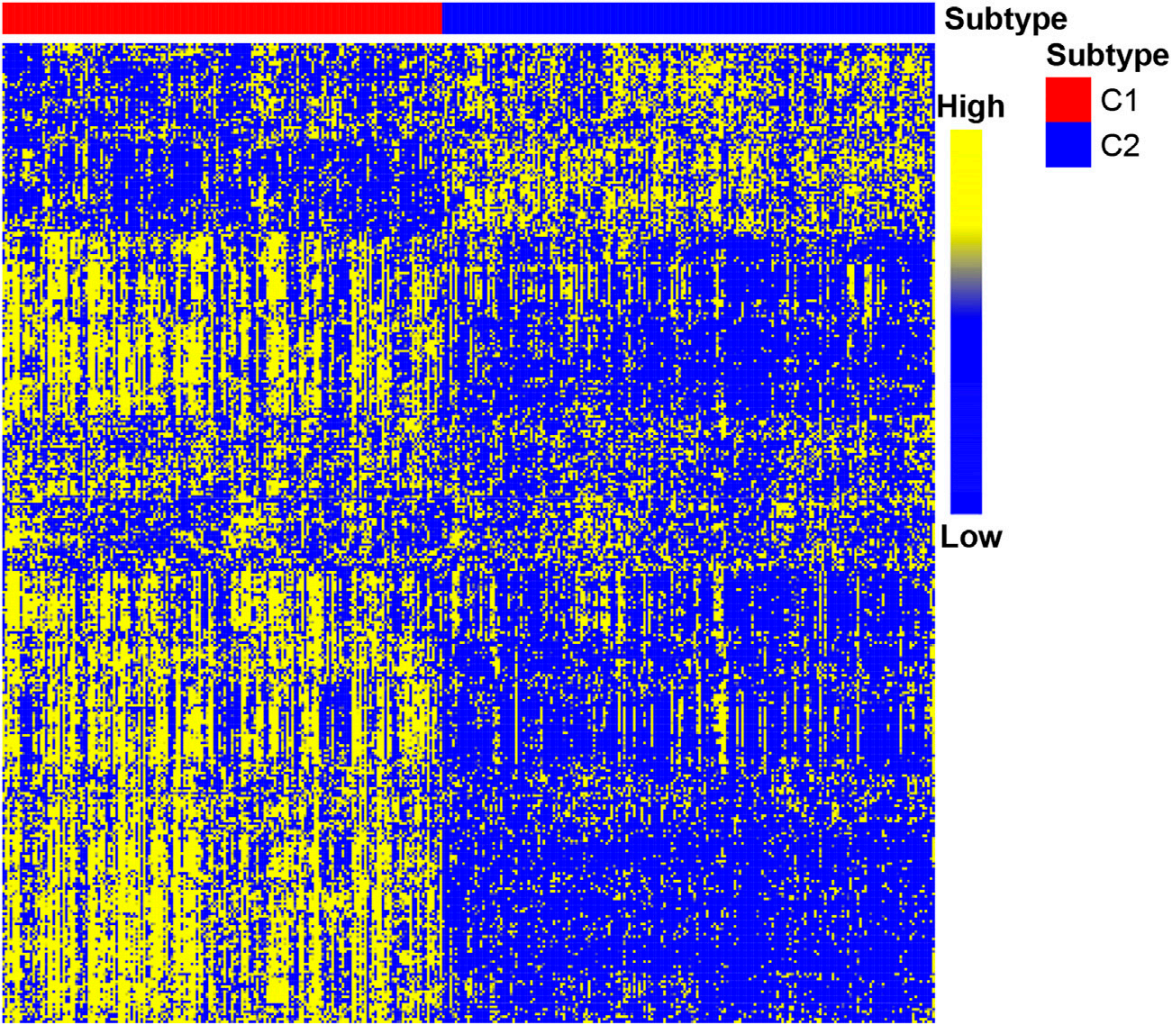
Heat map of C1/C2 in TCGA cohort. TCGA cohort was used as a discovery set, and 390 immune-related genes were selected for the development of subtypes. Data are presented in a matrix format, in which row represents an individual gene and each column represents a sample. The color in the cells reflects relatively high (yellow) and low (blue) expression levels.

After determining the correlation with prognosis, 390 genes were chosen to be used to define subtype (Supplementary Table S2). TCGA cohort was used as a discovery set, and four other cohorts (GSE62254 cohort; GSE84437 cohort; GSE15459 cohort; Tianjin cohort) were used as the validation sets. The clustering metrics showed that the optimal cluster number is 2 among these five datasets (Supplementary Figure S1). These two clusters were regarded as two subtypes of gastric cancer, which are called C1 and C2. The heat map of C1 and C2 is shown in Figure 2, which illustrated that the immune gene expression of C1/C2 was significantly different in TCGA cohort. The C1/C2 subtype was compared with other GC microenvironment signatures. The results of Fisher’s exact test illustrate that C1/C2 has association with TME signature and immune landscape subtypes in TCGA cohort, and C1/C2 has association with ACRG subtypes in GSE62254 cohort (*p* < 0.05) (Cristescu et al., 2015; Thorsson et al., 2019; Zeng et al., 2019) (Supplementary Figure S2).

### C1/C2 Predict the Survival of Gastric Cancer

**FIGURE 3:**
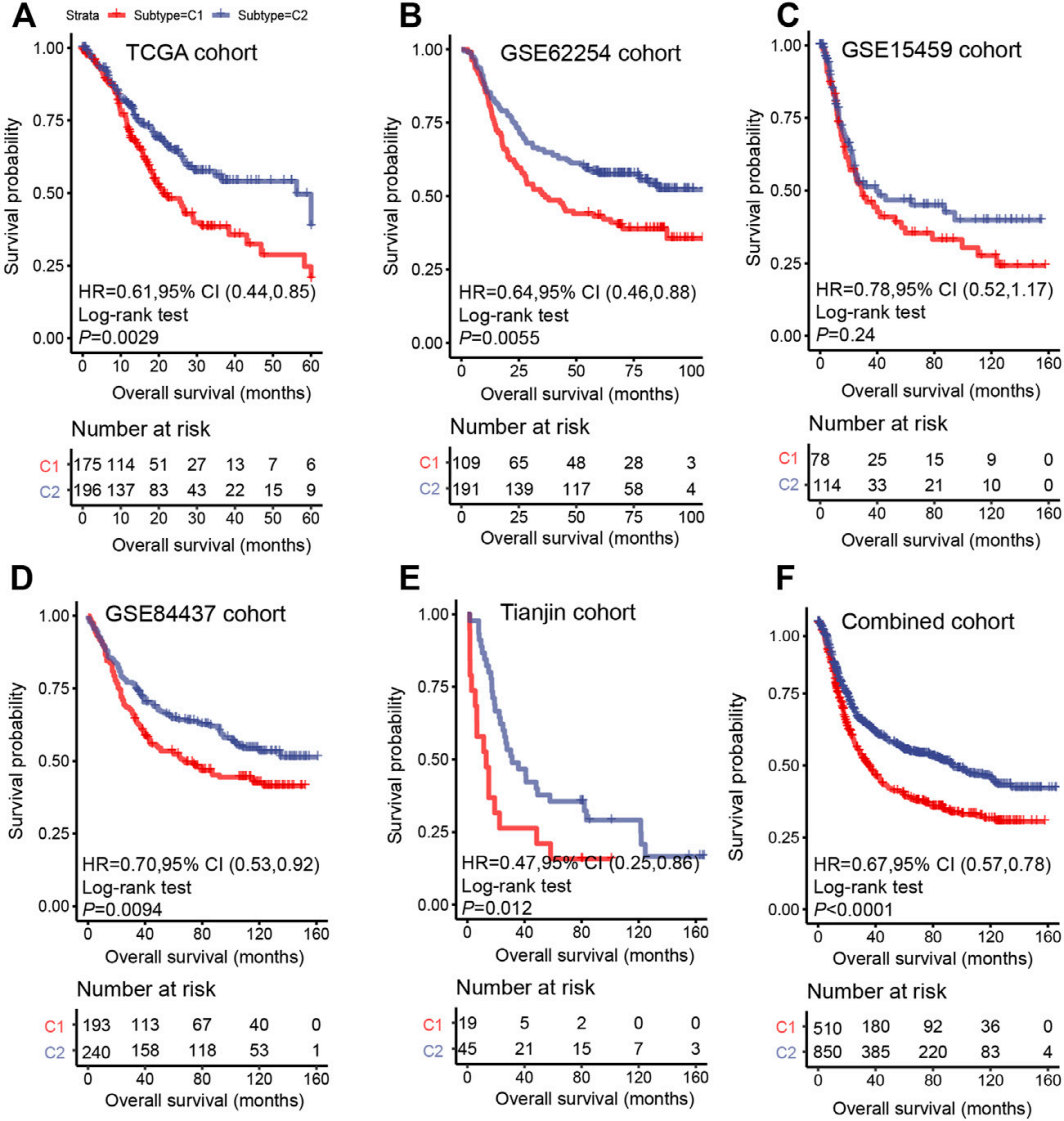
Survival analysis for C1/C2 subtype in **(A)** TCGA cohort, **(B)** GSE62254 cohort, **(C)** GSE15459 cohort, **(D)** GSE84437 cohort, **(E)** Tianjin cohort and **(F)** Combined cohort in C1/C2. Kaplan–Meier plots of overall survival (OS) among patients stratified by C1/C2. Hazard ratio (HR) was calculated by Cox regression analysis. A *p* value was obtained using the log-rank test.

**FIGURE 4:**
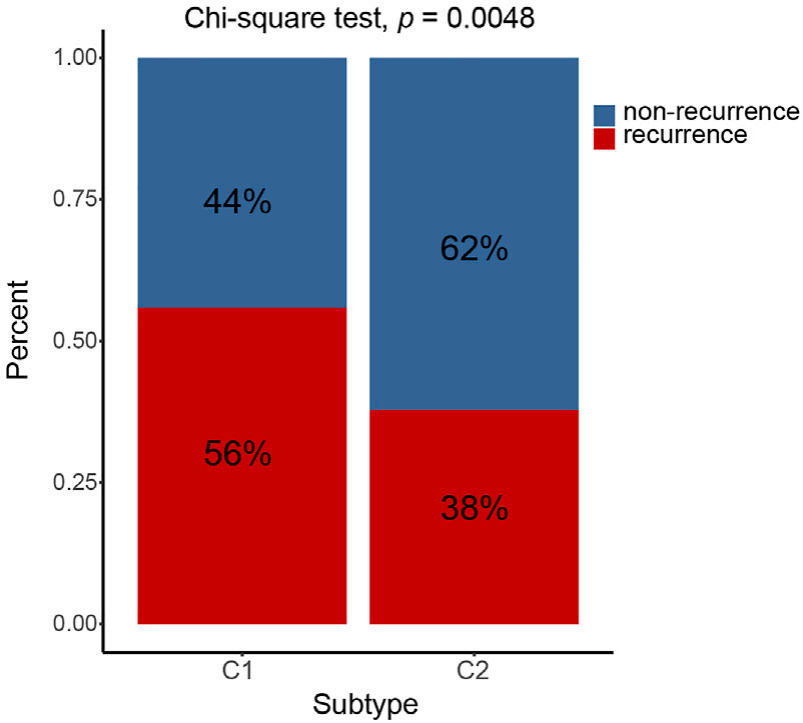
C1/C2 has different recurrence rates in GSE62254 cohort. In GSE62254 cohort, C2 has lower recurrence rate (38%) than C1 (56%). The chi-square test results showed a significant difference in the recurrence rate of C1/C2 (*p* = 0.0048).

The prognosis of C2 is significantly better than that of C1 in TCGA (HR = 0.61, 95% CI: 0.44–0.85, log-rank test: *p* = 0.0029), GSE62254 (HR = 0.64, 95% CI: 0.46–0.88, log-rank test, *p* = 0.0055), GSE84437 (HR = 0.70, 95% CI: 0.53–0.92, log-rank test, *p* = 0.0094),Tianjin (HR = 0.47, 95% CI: 0.25–0.86, log-rank test: *p* = 0.012), and combined cohorts (HR = 0.67, 95% CI: 0.57–0.78, log-rank test: *p* < 0.0001) (Figures 3A,B,D–F). The prognosis with C1 and C2 in the GSE15459 cohort has the same trend as other cohorts (GSE15459 cohort, HR = 0.78, 95% CI: 0.52–1.17, log-rank test, *p* = 0.24) (Figure 3C). In the GSE62254 cohort, C2 has lower recurrence rate (38%) than C1 (56%) (Figure 4). The chi-square test results showed a significant difference in the recurrence rate of C1/C2 (*p* = 0.0048).

**FIGURE 5:**
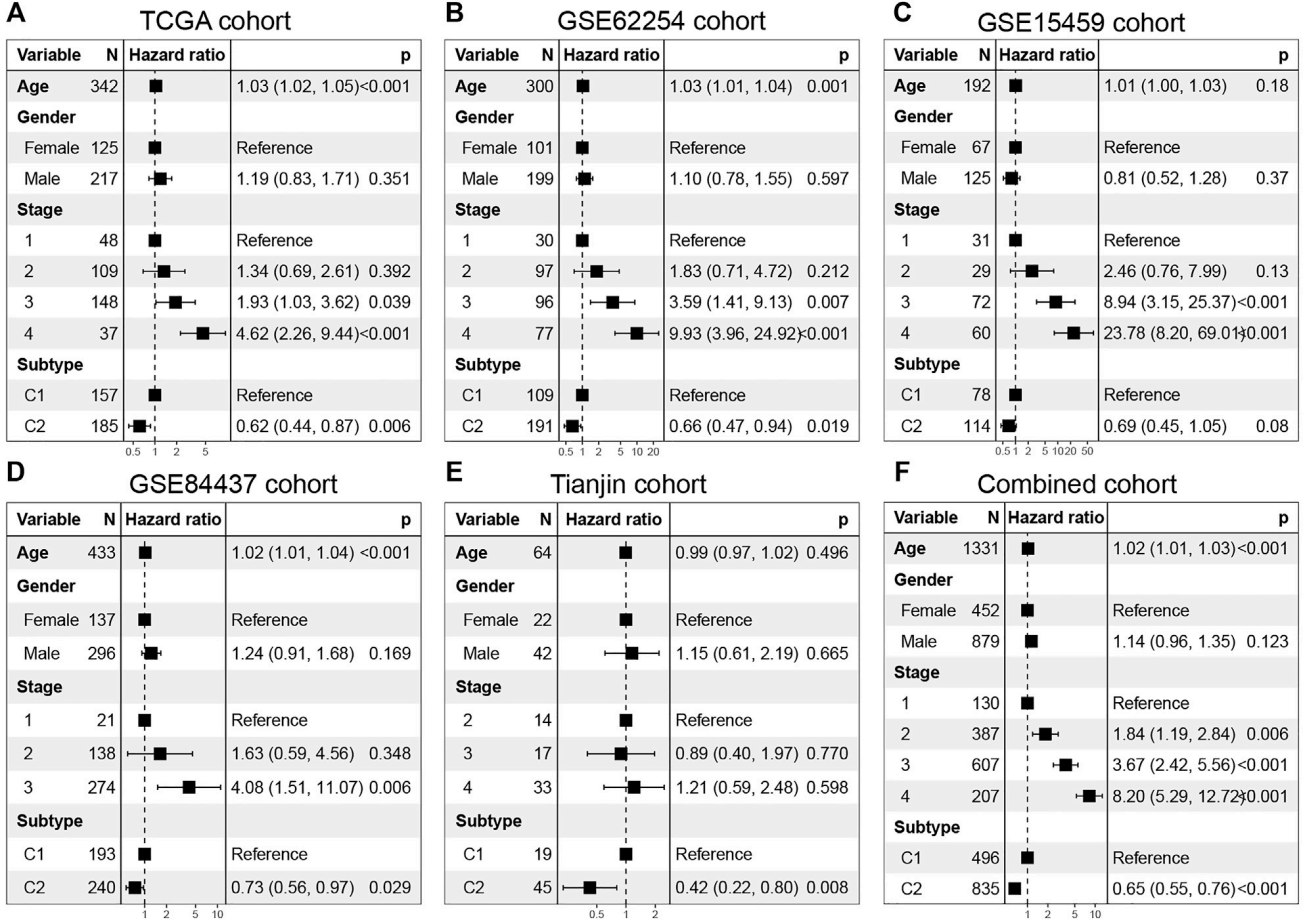
Multivarate Cox regression analysis of C1/C2 subtype in **(A)** TCGA cohort, **(B)** GSE62254 cohort, **(C)** GSE15459 cohort, **(D)** GSE84437 cohort, **(E)** Tianjin cohort and **(F)** Combined cohort. Multivariable Cox regression analysis was used to evaluate independent prognostic factors associated with overall survival, including age, gender, and tumor stage. A *p* value of less than 0.05 was considered statistically significant.

In addition, multivariable Cox regression in discovery set and validation set demonstrates that together with age, gender, and stage, C1 and C2 are still independent prognostic factors (TCGA cohort, HR = 0.62, 95% CI: 0.44–0.87, log-rank test: *p* = 0.006; GSE62254 cohort, HR = 0.66, 95% CI: 0.47–0.97, log-rank test, *p* = 0.019; GSE15459 cohort, HR = 0.69, 95% CI: 0.45–1.05, log-rank test, *p* = 0.08; GSE84437 cohort, HR = 0.73, 95% CI: 0.56–0.97, log-rank test, *p* = 0.029; Tianjin cohort, HR = 0.42, 95% CI: 0.22–0.80, log-rank test: *p* = 0.008) (Figures 5A–E). In the combined cohort, the C2 subtype remained a better prognostic factor than the C1 subtype (Figure 5F).

**FIGURE 6:**
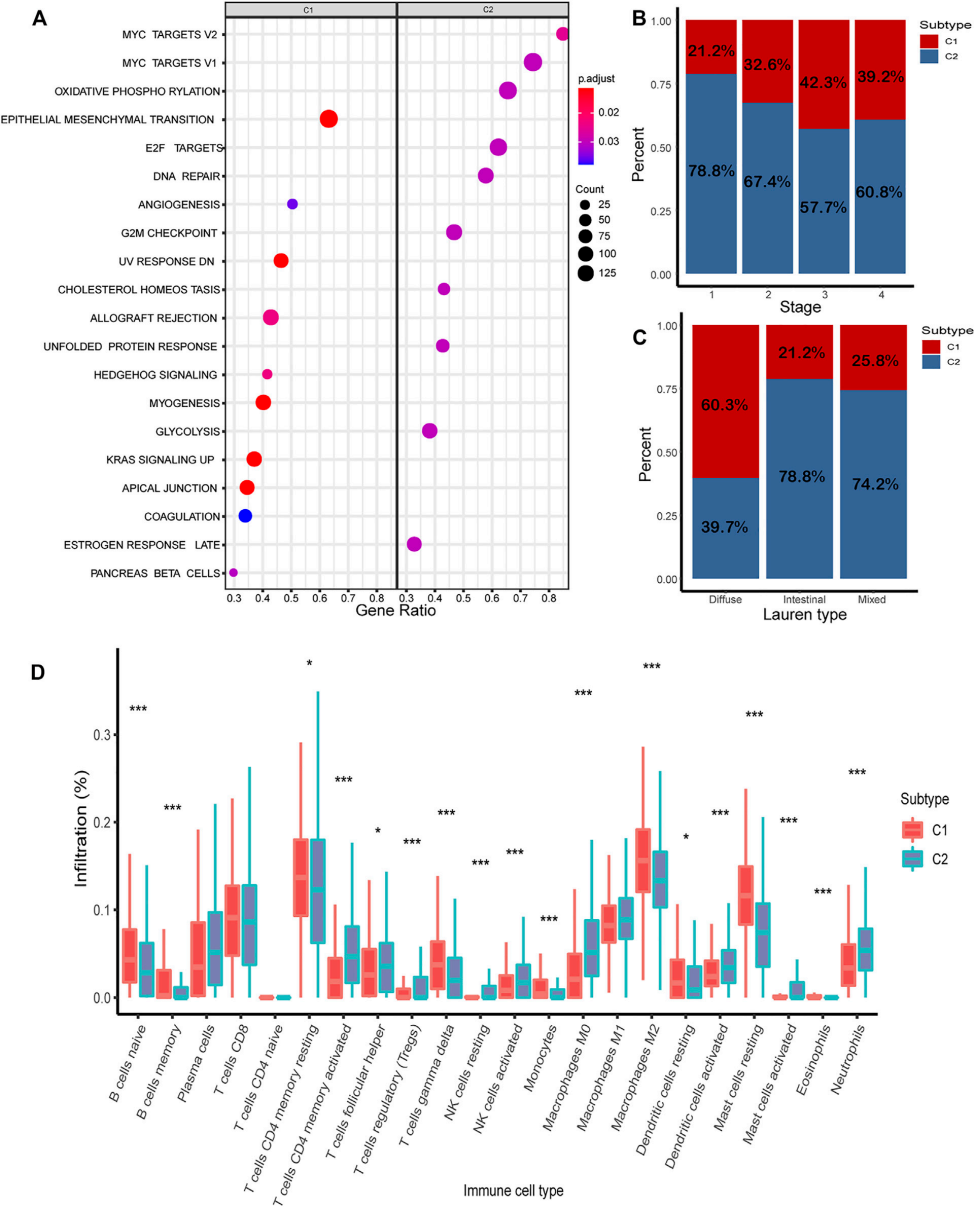
Analysis of biological characteristics of C1/C2. **(A)** Highly expressed genes in C1/C2 were enriched in 20 cancer-related pathways. **(B)** Proportion of C1/C2 in tumor stages 1–4. **(C)** Proportion of C1/C2 in Lauren type. **(D)** Result of CIBERSORT in C1/C2.

In the combined cohort, the proportion of C1/C2 in four stages was calculated. The proportion of C1 is relatively higher in advanced gastric cancer (stage 3: 42.3%; stage 4: 39.2%) (Figure 6B). The proportion of C1/C2 in the Lauren type in the GSE62254 and GSE15459 cohorts was calculated, and C1 accounted for 60.3%, 21.2, and 25.8%, respectively, in the diffuse type, intestinal type, and mixed type. It shows that a higher proportion of C1 was found in the diffuse type, which is the most malignant type (Figure 6C).

C1 and C2 successfully stratify patients by survival in several gastric cancer cohorts. It is also an independent prognosis factor. The results show the reproducibility and clinical significance of C1/C2.

### Biological Characteristics of C1/C2

The results of CIBERSORT demonstrated the immune infiltration of C1/C2 in the combined cohort. Most of the immune cells have significant differences between C1 and C2. In C2, such as T-cell CD4 memory activated, NK cells activated, mast cells activated, and dendritic cells activated, the composition of all these four kinds of activated immune cells was significantly higher than C1 (*p* < 0.05). In contrast, in C1, such as B cells were naive, T-cell memory resting, dendritic cells resting, and mast cells resting, and these kinds of immune resting cells were significantly higher than C2 (*p* < 0.05) (Figure 6D).

To further investigate the potential biological behavior of the molecular subtype, the DEGs were used for pathway enrichment in the combined cohort (Supplementary Table S3). Finally, 20 cancer-related pathways were enriched (Figure 6A). Genes highly expressed in C1 were enriched in “Epithelial Mesenchymal Transition,” “Angiogenesis,” and “UV Response.” Genes highly expressed in C2 were enriched in “MYC Target,” “Oxidative Phosphorylation,” and “E2F Target.”

The driver gene mutation between C1 and C2 was compared in TCGA cohort. It was observed that C2 had significantly more mutation events than C1 in *APC* (*p* = 0.0024), *NBEA* (*p* = 0.0026), *PIC3CA* (*p* = 0.0114), *XIRP2* (*p* = 0.0131), RNF43 (*p* = 0.0211), *SMAD4* (*p* = 0.0369), *TP53* (*p* = 0.0398), *KRAS* (*p* = 0.043), and *BNCA* (*p* = 0.0459), while C1 has significantly more mutation events than C2 in *BNC2* (*p* = 0.0459), *CDH1* (*p* = 0.0488), and *CTNNB1* (*p* = 0.05). The results showed differences in driver genes mutation of C1 and C2 (Supplementary Table S1). The tumor mutation burden (TMB) was also calculated, where C2 has higher TMB than C1 (*p* < 0.05) (Supplementary Figure S3).

## Discussion

The clinical significance of the molecular subtype has been demonstrated in many kinds of cancers. However, few researchers have used immune signatures to predict gastric cancer subtypes.

A major clinically relevant finding in this study is based on signature of 390 immune-related genes; we classify gastric cancer into two prognostically distinct subgroups, namely, C1 and C2. The prognostic significance of C1/C2 was independent of age, gender, and stage. The C1 subtype is featured by “Epithelial Mesenchymal Transition (EMT)” and “Angiogenesis,” which had poorer overall survival, whereas the C2 subtype is characterized by “MYC Target,” “Oxidative Phosphorylation,” and “E2F Target,” with better overall survival than those in C1. Notably, previous research studies reported that EMT was shown to strongly enhance cancer cell motility and dissemination; it plays an important role in cancer metastasis (Brabletz, 2012; Brabletz et al., 2018). Angiogenesis is essential for the late stages of carcinogenesis, allowing the tumor to grow beyond 1–2 mm in diameter; it is associated with the malignancy of tumor (Sharma et al., 2001; Albini et al., 2012). Such processes may cause poor survival in C1.

The results of CIBERSORT suggest that C1 and C2 have very different immune environments. In C2, such as CD4 memory activated, NK cells activated, mast cells activated, and dendritic cells activated, and the composition of all these four kinds of activated immune cells are significantly higher than C1. In contrast, in C1, such as B cells were naive, T cells memory resting, dendritic cells (DC) resting, and mast cells resting, and these kinds of immune resting cells significantly higher than C2. The microenvironment between C1 and C2 shows a marked difference. Previous research studies reported that memory CD4 T cells could make effector cytokines early in response and they could enhance B-cell and CD8 T-cell responses, which enhance immune response (MacLeod et al., 2009). NK cells are important immune cells; they could swiftly kill multiple adjacent cells which show surface markers associated with oncogenic transformation; and they could also magnify immune responses (Shimasaki et al., 2020). Mast cells are evolutionarily ancient cells, and they finely modulate not only immune responses but also the mechanism of several inflammatory disorders, including cancer, autoimmunity, and infection (Frossi et al., 2017). DCs are a diverse group of specialized antigen-presenting cells; they play key roles in the initiation and regulation of innate and adaptive immune responses (Wculek et al., 2020). The activation of these immune cells in C2 may indicate higher immune activity, which leads better prognosis. The resting of these immune cells may cause poor immune activity in C1, which leads to worse prognosis in C1.

The proportion of C1/C2 in four tumor stages demonstrates C1 has higher proportion in advanced gastric cancer, while C2 has higher proportion in early stages. The proportion of C1/C2 in the Lauren type shows that C1 has the highest proportion in the diffuse type, while C2 has higher proportion in the intestinal type. This indicates that C1 has some characteristics of malignant gastric cancer.

The somatic mutation event of SMGs shows significant differences between C1 and C2, and C2 has higher TMB than C1. Previous research reported that TMB can be used as an indicator to predict the response to immunotherapy, and patients with high TMB were observed to have better clinical outcomes (Gibney et al., 2016; Gandara et al., 2018; Mandal et al., 2019). It also reported that high TMB is associated with a better prognosis in gastric cancer (Cai et al., 2020; Wang et al., 2021). The differences in mutation characteristics may lead to different clinical outcomes of C1/C2, and it also could offer some new insights into immunotherapy in gastric cancer.

In total, in this research, we predict C1 and C2, two subtypes of gastric cancer. Much evidence has shown that there are many different biological characteristics between C1 and C2. It makes two subtypes that could predict prognosis in gastric cancer patients. However, this research still has some limitation. First, the sample size is not large enough; therefore, research may not cover all types of gastric cancer. Second, due to the lack of clinical data, the subtypes in this research could only be used to predict the survival of gastric cancer patients but could not predict their response to chemoradiotherapy and immunotherapy. If more gastric cancer chemotherapy and immunotherapy data could be combined, C1/C2 could be given more clinical significance and immune characteristics could provide more insights into gastric cancer treatment.

Our research has developed two molecular subtypes of gastric cancer, and we have analyzed their immune signature and biological function. These findings may offer some new knowledge of molecular mechanisms for study on treatment of gastric cancer.

## Data Availability

The original contributions presented in the study are included in the article/Supplementary Material, further inquiries can be directed to the corresponding authors.

## Supplementary Material

Supplementary Figure S1 The CNMF results of five cohorts and combined cohort. The ordinate “Cophenetic” means cophenetic correlation coefficient. We select the rank value where the magnitude of the cophenetic correlation coefficient begins to fall. The ordinate “Dispersion” between 0 and 1 measures the class assignments’ robustness concerning random initial conditions, closing to 0 or 1 means the model's stability. In five cohorts and combined cohort, 2 is the best number of clusters.

Supplementary Figure S2 Association between molecular subtype C1/C2 and tumor microenvironment signature. **(A)** Association between C1/C2 and TME signature in TCGA cohort. **(B)** Association between C1/C2 and immune landscape subtype in TCGA cohort. **(C)** Association between C1/C2 and ACRG subtype in GSE62254 cohort

Supplementary Figure S3 TMB of C1/C2 in TCGA cohort. TMB of TCGA cohort was calculated. In this part, TMB equal to mutation events occur in each sample. TMB of C2 significantly higher than C1 (*p* <0.05).

Supplementary Table S1 Mutation prevalence of driver genes in C1 versus C2 in TCGA cohort.
