# Supplementary figures and images for: Prediction of Two Molecular Subtypes of Gastric Cancer Based on Immune Signature
Source: Front Genet. 2022 Jan 17;12:793494. doi: 10.3389/fgene.2021.793494 (PMC8802764; doi:10.3389/fgene.2021.793494)

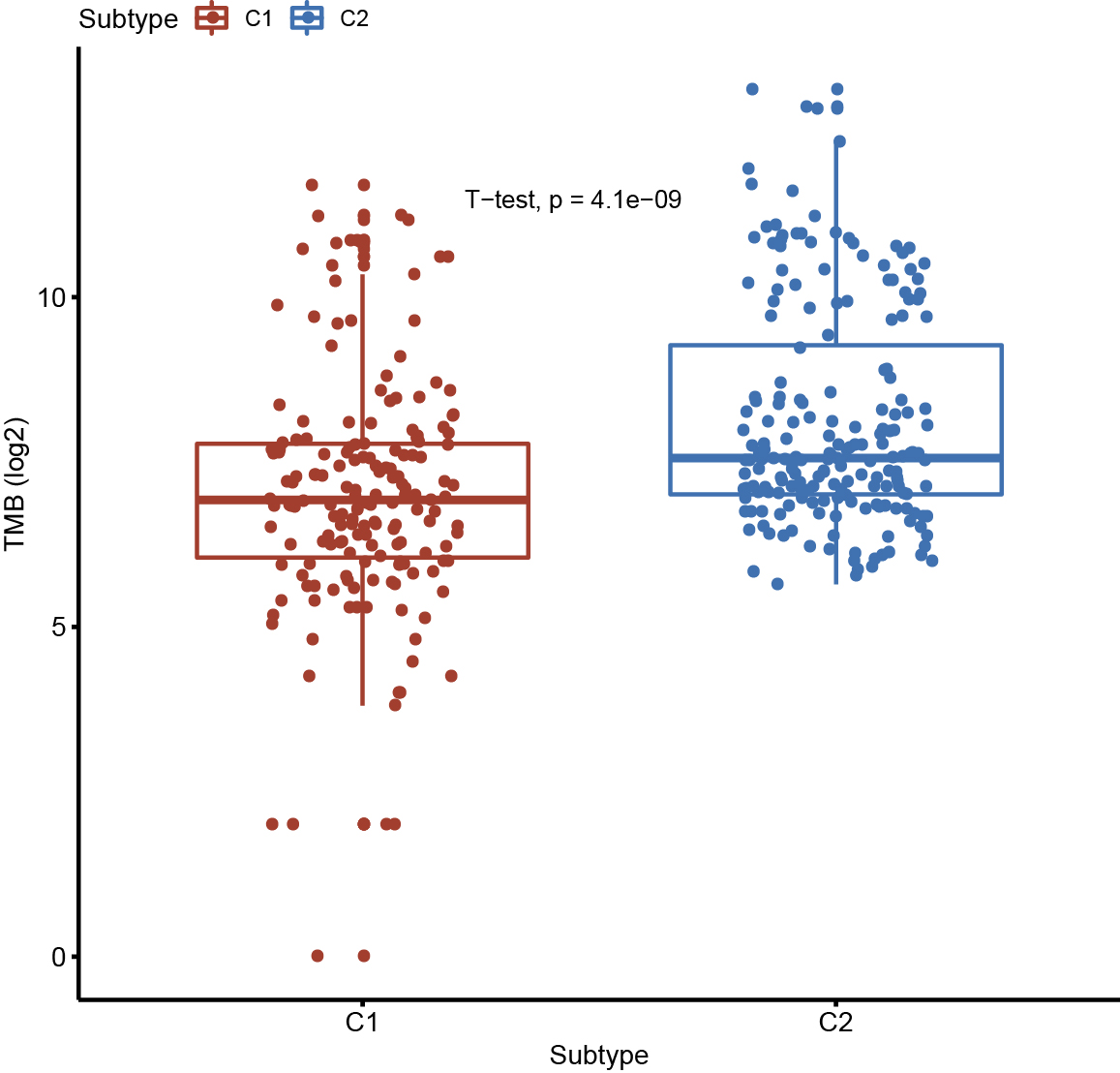

Supplement: Supplementary file 3 [file Image3.jpg]

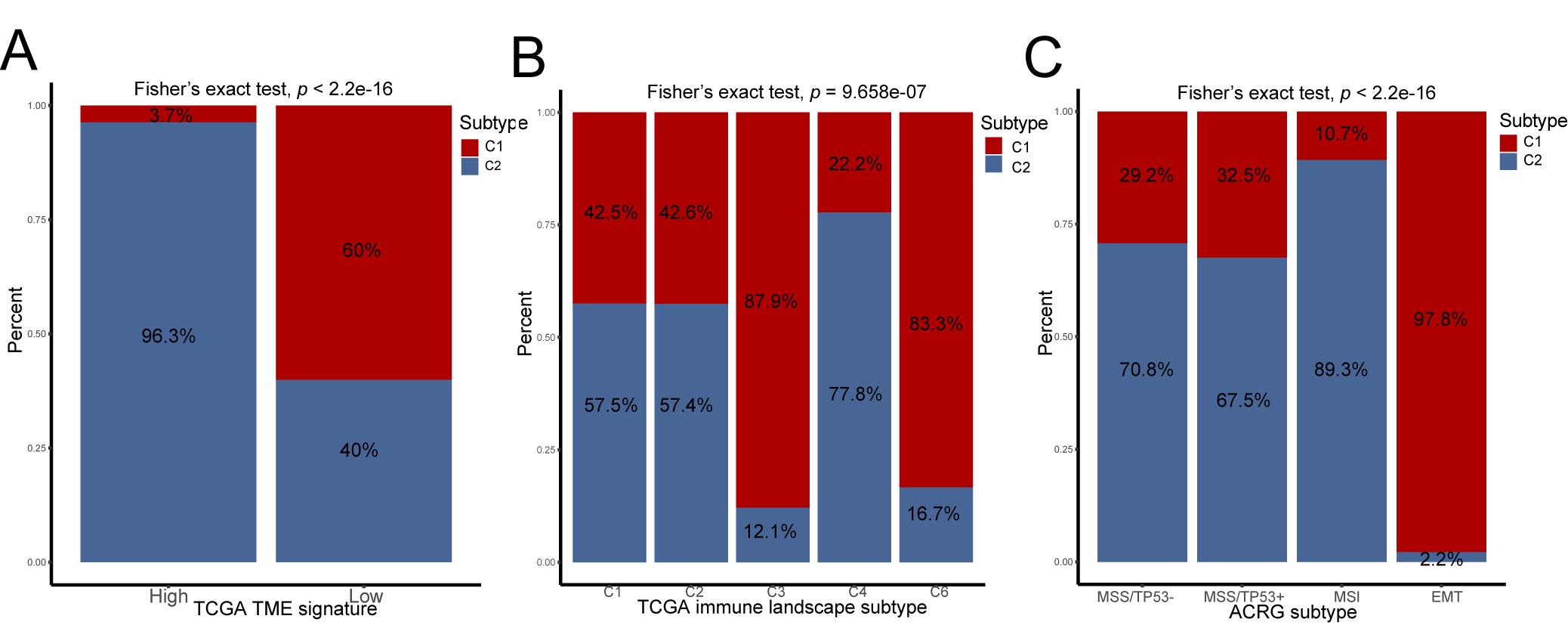

Supplement: Supplementary file 4 [file Image2.tif]

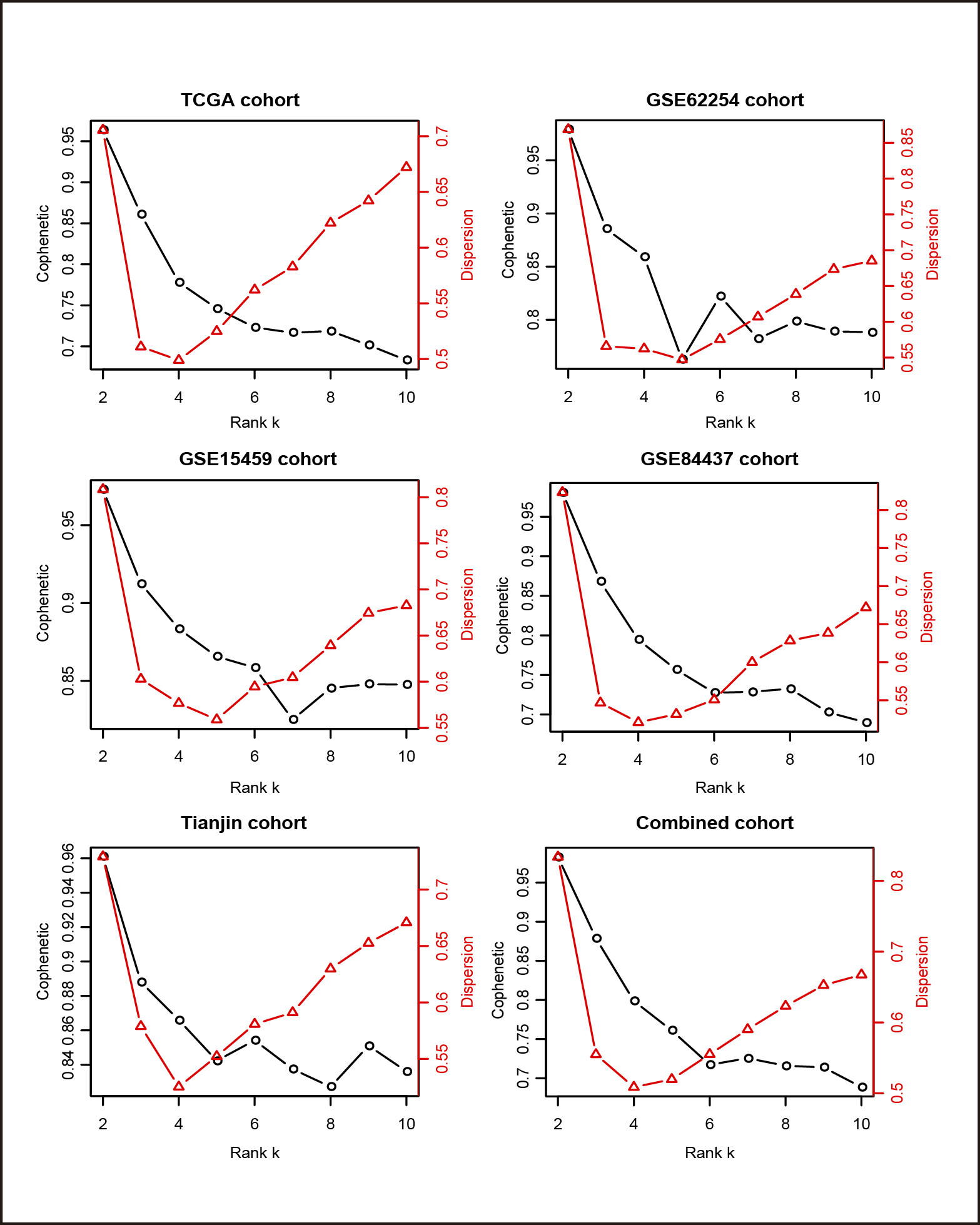

Supplement: Supplementary file 6 [file Image1.jpg]
